# Thoracoscopic epicardial left ventricular bipolar lead implantation with the use of automated titanium fasteners (Cor-Knot®)

**DOI:** 10.1186/s13019-019-0945-4

**Published:** 2019-06-28

**Authors:** Thibault Schaeffer, Constantin Mork, Joachim Erb, Oliver Reuthebuch

**Affiliations:** 1 Department of cardiac surgery, University Hospital of Basel, Spitalstrasse 31, CH 4031 Basel, Switzerland; 2 Department of anesthesiology, University Hospital of Basel, Basel, Switzerland

**Keywords:** Thoracoscopy, Epicardial pacemaker electrode, Cor-knot

## Abstract

**Background:**

Pacemaker implantation techniques using thoracoscopy have been described since about 25 years. However, the published reports concerning types of electrodes refer mostly to monopolar screw-in leads. We report our experience of thoracoscopic implantation of a bipolar suture-on epicardial electrode with monofilamentous sutures tightened by automated fasteners to avoid hand-tied knots.

**Case presentation:**

A 69-year-old Caucasian female patient with a cardiac resynchronization therapy – defibrillator (CRT-D) due to dilated cardiomyopathy required the implantation of a supplementary left ventricluar resynchronization electrode. Because of unfavorable venous access, we chose a thoracoscopic approach. A bipolar suture-on epicardial electrode, was implanted by means of polypropylene monofilament 2–0 threads and automated titanium fasteners (Cor-Knot®). The intervention was uneventful. The correct function of the device was confirmed postoperatively and the patient was dismissed within 3 days from hospital. Six months after implantation the cardiologic control asserted regular device function and restitution of normal ejection fraction (EF 60%).

**Conclusion:**

This case demonstrates the feasibility, safety and effectiveness of automated fasteners in the setting of thoracoscopic implantation of epicardial bipolar suture-on leads.

## Background

The implantation of cardiac resynchronization therapy (CRT) leads for the management of cardiac dyssynchrony is generally performed percutaneously via the coronary sinus or a left cardiac vein. For various reasons (anatomic variation, venous stricture or thrombosis) the vascular access can be hampered. Alternatively, an epicardial electrode can be fixed on the free wall of the left ventricle. In order to minimize the surgical trauma, pacemaker implantation techniques using thoracoscopy have been described since the early 1990’s [1, 2]. However, in these cases predominantly monopolar screw-in leads were used [3]. We report our experience of thoracoscopically implanting a bipolar suture-on epicardial electrode with monofilamentous sutures tightened with automated fasteners to avoid hand-tied knots.

### Case presentation

A 69-year-old Caucasian female patient (born male) was diagnosed with dilated cardiomyopathy and had undergone the implantation of a CRT-Internal Cardiac Defibrillator (CRT-ICD Visionist®, Boston Scientific Corporation, USA) in 2009. Because of inopportune stimulation of the diaphragm, the coronary sinus electrode had to be deactivated. In 2018, a newly inserted electrode failed to achieve resynchronization, presumably due to an excessive lateral position. However, an additional electrode insertion was not technically feasible due to venous obstruction. Thus, the patient was referred to surgery for a minimal-invasive left ventricular epicardial lead implantation.

**Fig. 1:**
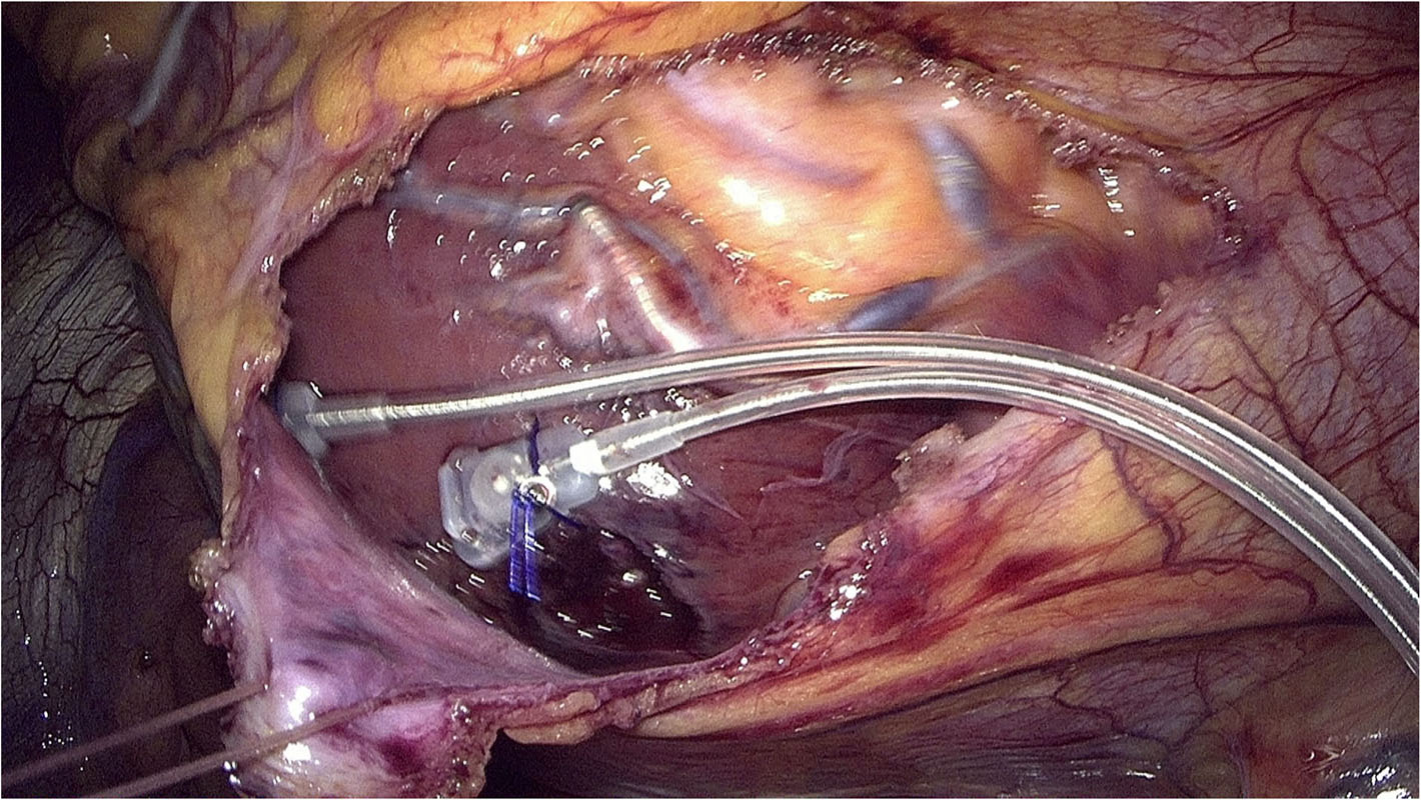
bipolar left ventricular lead (CAPSURE EPI 4968–35 cm, Medtronic®, USA) fixed on the beating myocardium by means of Cor-Knot®

The operation was performed thoracoscopically. Three trocars were used: two 12 mm trocars in the 4th and 5th intercostal space on the anterior axillary line (thoracoscope, grasper), and one 7 mm trocar via the pouch of the CRT device (hook, needle holder). CO_2_ insufflation (15 mmHg) was used for better visibility. A pericardial window was dissected ventral to the left phrenic nerve. The two coils of the epicardial bipolar electrode (CAPSURE EPI 4968–35 cm, Medtronic®, USA) were placed on the lateral wall of the beating heart. To safely pass the myocardium without tearing, a monofilament 2–0 polypropylene suture was used. Further, to avoid extracorporeal hand knotting leading to potential tension on the suture and probable disruption of the fragile myocardium, automated titanium fasteners (Cor-Knot®, LSI Solutions, USA) were utilized (Fig. 1). After testing, the electrode was passed into the pouch and connected to the CRT device. The pericardium was closed with braided sutures and further titanium fasteners. A pleural aspiration drain was inserted before the left lung was re-expanded and the wounds were closed with absorbable sutures.

The pacemaker interrogation attested the correct function of the implanted lead (pacing threshold 0.70 V by 0.5 ms; sensing threshold: 10 mV; impedance: 582 Ω). The drain was removed after 48 h and the patient was dismissed from the hospital without complication on the third postoperative day. Cardiologic work-up 6 months after implantation confirmed correct device performance with 100% resynchronization rate and an expected battery life of 11 years. Clinically the patient was cardiopulmonary compensated with a complete restitution of left ventricular ejection fraction (EF 60%).

## Discussion and conclusions

Several studies have shown that bipolar leads have superior sensing and pacing thresholds upon monopolar leads over time [4–6]. The use of Cor-Knot® is indicated for approximation of soft tissue and prosthetic materials in conjunction with 2–0 polyester or 2–0 polypropylene (https://www.lsisolutions.com/). We presume several advantages in using titanium fasteners: first, it lowers the risk of tearing out the myocardium as if hand-tying the knot with knot-pushers. Second, it simplifies the procedure by avoiding the tangling of long sutures in the trocar or the uncontrolled slipping of the electrode while tying. Third, it significantly accelerates the procedure. Finally, it reduces the time for CO_2_ pneumothorax and thus the potential mechanical compression of hearts with impaired function. To our knowledge this is the first case of using this fixation-technique. In view of the satisfying result, we will apply this method in future implantations.

We conclude, that with the use of automated fasteners (Cor-Knot®), the thoracoscopic implantation of epicardial bipolar leads appears to be feasible, fast, safe and effective compared to hand-tied knots.

## Data Availability

The datasets used and/or analysed during the current study are available from the corresponding author on reasonable request.

## Abbreviations

CRT: Cardiac resynchronization therapy
CRT-D: Cardiac resynchronization therapy defibrillator
CRT-ICD: Cardiac resynchronization therapy – implantable cardioverter defibrillator
